# Supplementary figures and images for: When to use one-dimensional, two-dimensional, and Shifted Transversal Design pooling in mycotoxin screening
Source: PLoS One. 2020 Aug 5;15(8):e0236668. doi: 10.1371/journal.pone.0236668 (PMC7406063; doi:10.1371/journal.pone.0236668)

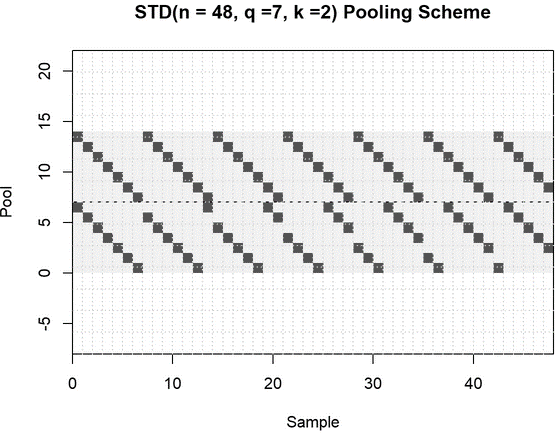

Supplement: S1 Fig — The horizontal dashed lines split the pooling scheme into 2 layers. There are 14 pools; each layer contains 7 pools and each pool comprises a combination of samples indicated as squares. (TIF) [file pone.0236668.s001.tif]

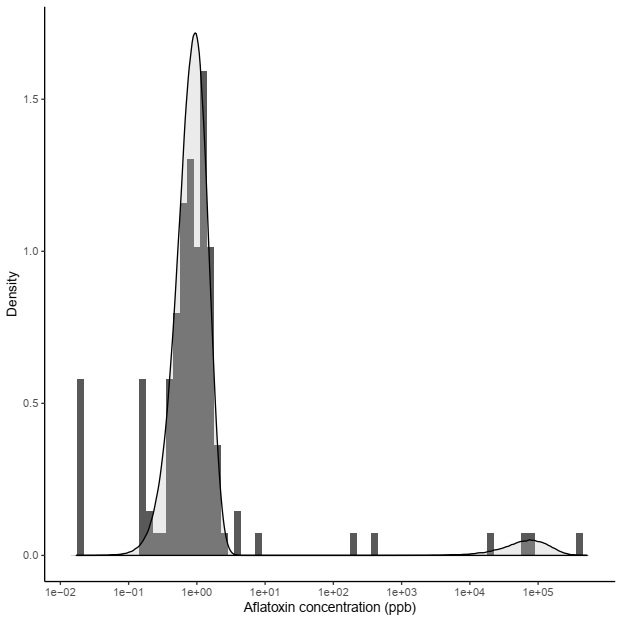

Supplement: S2 Fig — The histogram represented the real aflatoxin concentration distribution, with 6 kernels (4%) ≥ 20 ppb aflatoxin and 132 kernels (96%) < 20 ppb aflatoxin. The density plot (grey shaded area) illustrated the distribution of simulated data with 9.6 × 10^5^ healthy kernels (96%) and 4 × 10^4^ contaminated kernels (4%). (TIF) [file pone.0236668.s002.tif]

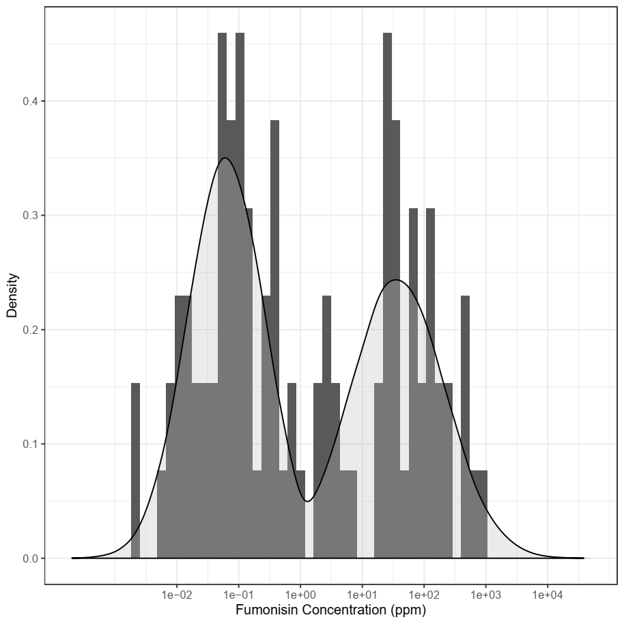

Supplement: S3 Fig — The histogram represented the experimental fumonisin concentration with 43 kernels (46%) ≥ 1 ppm and 50 kernels (54%) < 1 ppm. The density plot (grey shaded area) illustrated the distribution of simulated data with 5.4 × 10^5^ (54%) healthy kernels and 4.6 × 10^5^ (46%) contaminated kernels. (TIF) [file pone.0236668.s003.tif]

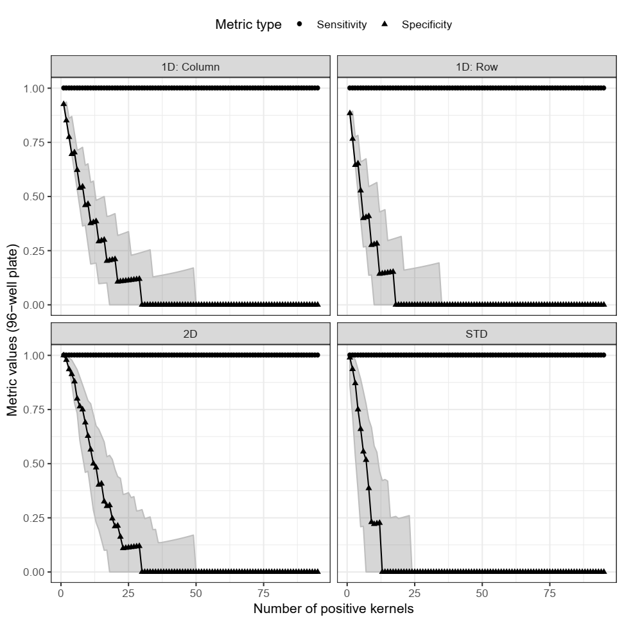

Supplement: S4 Fig — The point represents the median and the shaded area represents the range from 2.5th percentile to 97.5th percentile. Top left panel is 1D pooling where columns are pooled, top right panel is 1D pooling where rows are pooled, bottom left panel is 2D pooling, and bottom right panel is STD-pooling (n = 96; q = 5; k = 3). (TIF) [file pone.0236668.s004.tif]

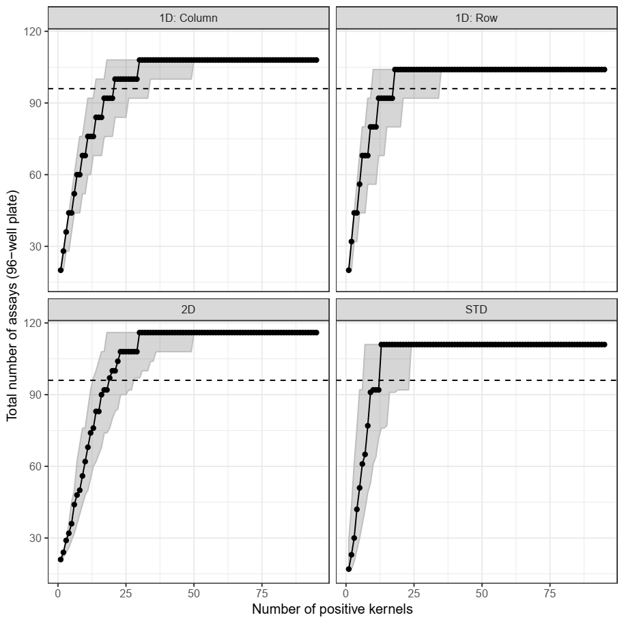

Supplement: S5 Fig — The dashed line indicates that 96 tests would be needed without pooling. The dot represents the median and the shaded area represents the range from 2.5th percentile to 97.5th percentile. Top left panel is 1D pooling where columns are pooled, top right panel is 1D pooling where rows are pooled, bottom left panel is 2D pooling, and bottom right panel is STD-pooling (n = 96; q = 5; k = 3). (TIF) [file pone.0236668.s005.tif]

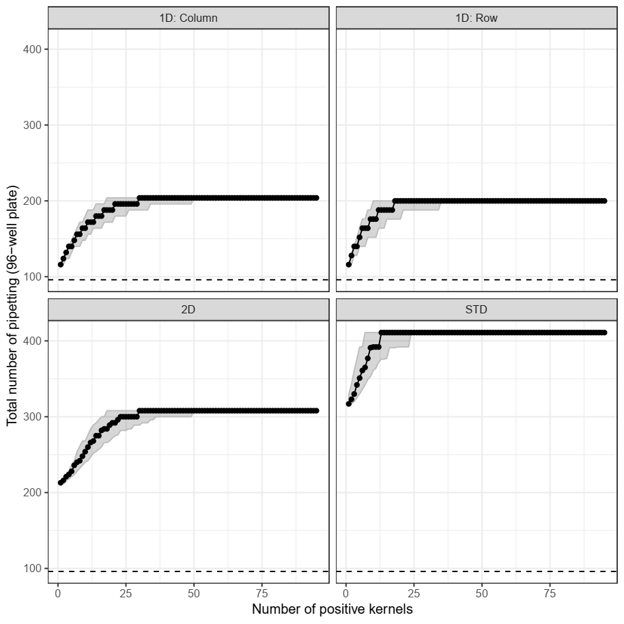

Supplement: S6 Fig — The dashed line indicates that without pooling 96 times of pipetting would be needed to transfer samples into ELISA assay plate. The dot represents the median and the shaded area represents the range from 2.5th percentile to 97.5th percentile. Top left panel is 1D pooling where columns are pooled, top right panel is 1D pooling where rows are pooled, bottom left panel is 2D pooling, and bottom right panel is STD-pooling (n = 96; q = 5; k = 3). (TIF) [file pone.0236668.s006.tif]
