# Supplementary material for: When to use one-dimensional, two-dimensional, and Shifted Transversal Design pooling in mycotoxin screening
Source: PLoS One. 2020 Aug 5;15(8):e0236668. doi: 10.1371/journal.pone.0236668 (PMC7406063; doi:10.1371/journal.pone.0236668)
Supplement: S1 Table — Each pool consists of 7 samples of equal volume. Number 1 to 48 represents the sample index and number 0 represents the solvent (80% methanol solution). (DOCX) [file pone.0236668.s007.docx]

**S1 Table. The STD-pooling scheme (n = 48; q = 7; k = 2) in table format for 48 samples.** Each pool consists of 7 samples of equal volume. Number 1 to 48 represents the sample index and number 0 represents the solvent (80% methanol solution).

|  | Sample A | Sample B | Sample C | Sample D | Sample E | Sample F | Sample G |
| --- | --- | --- | --- | --- | --- | --- | --- |
| Pool 1 | 1 | 8 | 15 | 22 | 29 | 36 | 43 |
| Pool 2 | 2 | 9 | 16 | 23 | 30 | 37 | 44 |
| Pool 3 | 3 | 10 | 17 | 24 | 31 | 38 | 45 |
| Pool 4 | 4 | 11 | 18 | 25 | 32 | 39 | 46 |
| Pool 5 | 5 | 12 | 19 | 26 | 33 | 40 | 47 |
| Pool 6 | 6 | 13 | 20 | 27 | 34 | 41 | 48 |
| Pool 7 | 7 | 14 | 21 | 28 | 35 | 42 | 0 |
| Pool 8 | 1 | 14 | 20 | 26 | 32 | 38 | 44 |
| Pool 9 | 2 | 8 | 21 | 27 | 33 | 39 | 45 |
| Pool 10 | 3 | 9 | 15 | 28 | 34 | 40 | 46 |
| Pool 11 | 4 | 10 | 16 | 22 | 35 | 41 | 47 |
| Pool 12 | 5 | 11 | 17 | 23 | 29 | 42 | 48 |
| Pool 13 | 6 | 12 | 18 | 24 | 30 | 36 | 0 |
| Pool 14 | 7 | 13 | 19 | 25 | 31 | 37 | 43 |
