# Supplementary material for: When to use one-dimensional, two-dimensional, and Shifted Transversal Design pooling in mycotoxin screening
Source: PLoS One. 2020 Aug 5;15(8):e0236668. doi: 10.1371/journal.pone.0236668 (PMC7406063; doi:10.1371/journal.pone.0236668)
Supplement: S2 Table — (DOCX) [file pone.0236668.s008.docx]

**S2 Table. Glossary of important Shifted Transversal Design parameters.**

| Parameters | Type | Definition |
| --- | --- | --- |
| $n$ | Input | Sample size |
| $E$ | Input | Maximum number of errors expected |
| $m$ | Input | Maximum number of samples allowed to mix in one pool |
| $d$ | Input | Maximum number of positive samples expected |
| $q$ | Derived | Number of pools per layer |
| $k$ | Derived | Number of layers |
| $t$ | Derived | Number of pools in total |
