# Supplementary material for: When to use one-dimensional, two-dimensional, and Shifted Transversal Design pooling in mycotoxin screening
Source: PLoS One. 2020 Aug 5;15(8):e0236668. doi: 10.1371/journal.pone.0236668 (PMC7406063; doi:10.1371/journal.pone.0236668)
Supplement: S1 Appendix — (DOCX) [file pone.0236668.s009.docx]

# S1 Appendix

Construction of the STD-pooling scheme with $n$ = 48, $q$ = 7, and $k$ = 2 was based on the practical instruction from (Kainkaryam & Woolf, 2008) and is reproduced here. Detailed mathematical proofs could be found in (Thierry-Mieg, 2006).

## 1. Determination of STD-pooling parameters

Input parameters: $n, d, E, m$

Derived parameters: $q, \Gamma, k, t$

1. Set the input parameters.

$n=$ 48, $d$ = 1, $E$ = 0, $m$ = 8.

1. Find all possible prime numbers $q$. Based on the rule of $q<n$, a list of 15 prime numbers (2, 3, 5, 7, 11, 13, 17, 19, 23, 29, 31, 37, 41, 43, 47) are considered candidates for $q$.
2. Find the compression power, $\Gamma=\left\lceil\frac{logn}{logq} \right\rceil-1$. Then set $k=d\times\Gamma+2\times E+1=d\times\Gamma+1$. A total of 15 pairs of ($q$, $k$) are formed.
3. Identify all the $\left( q, k \right)$ pairs that meet the inequality criterion: $k\leq q+1$.
4. Perform further inequality tests to determine the number of tests $t$. As all the $\left( q,k \right)$ pairs meet the criteria: $k<q+1, \left\lceil\frac{n}{q} \right\rceil\leq m$, the number of tests needed are set as $t=q\times k$.
5. Choose the $\left( q,k \right)$ pair that requires the least number of tests $t$. In this case, the pair ($q=7,k=2$) is chosen, which requires $t=14$ tests.

## 2. Construction of STD-pooling scheme (n = 48; q = 7; k = 2)

Let $M$ be a $14\times48$ boolean matrix, i.e. a matrix with 0’s and 1’s only. $L_{0}, L_{1}$are two $7\times48$ boolean matrices representing two layers. Each layer has 48 columns denoted as $C_{j,0}, C_{j,1},\ldots,C_{j,i},\ldots,C_{j,47}$, where $j$ = 0, 1, 2, 3. The relation between $L_{j}$ and $C_{j,i}$ is shown below.

$$L_{j}=\left[ \begin{matrix} C_{j,0} & C_{j,1} & \ldots\end{matrix}\begin{matrix} & C_{j,47} \end{matrix} \right]$$

Let $\sigma$ be a circular shift function. For any $x_{1}, \ldots,x_{7}$ that takes the value of either 0 or 1, applying $\sigma$ once on the vector $\left( x_{1}, x_{2}, \ldots, x_{7} \right)^{T}$ will shift all the elements downward by one unit. Applying $\sigma$ n times on the vector will then shift all the elements by n units as shown below.

$$\sigma\left[ \begin{matrix} x_{1} \\ x_{2} \\ \begin{matrix} \vdots\\ x_{7} \end{matrix} \end{matrix} \right]=\left[ \begin{matrix} x_{7} \\ x_{1} \\ \begin{matrix} \vdots\\ x_{6} \end{matrix} \end{matrix} \right], \sigma^{2}\left[ \begin{matrix} x_{1} \\ x_{2} \\ \begin{matrix} \vdots\\ x_{7} \end{matrix} \end{matrix} \right]=\left[ \begin{matrix} x_{6} \\ x_{7} \\ \begin{matrix} \vdots\\ x_{5} \end{matrix} \end{matrix} \right], \sigma^{7}\left[ \begin{matrix} x_{1} \\ x_{2} \\ \begin{matrix} \vdots\\ x_{7} \end{matrix} \end{matrix} \right]=\left[ \begin{matrix} x_{1} \\ x_{2} \\ \begin{matrix} \vdots\\ x_{7} \end{matrix} \end{matrix} \right]$$

Let the column $i$ from layer $j$ to be $C_{j,i}=\sigma^{s\left( i,j \right)}C_{0,0}$, where $i$ = 0, 1, …, 47,

$$C_{0,0}=\left[ \begin{matrix} 1 \\ 0 \\ \begin{matrix} \vdots\\ 0 \end{matrix} \end{matrix} \right], s\left( i,j \right)=\left\{ \begin{matrix} \sum_{c=0}^{\Gamma} j^{c}\left\lfloor\frac{i}{q^{c}} \right\rfloor, j<q \\ \left\lfloor\frac{i}{q^{\Gamma}} \right\rfloor, j=q \end{matrix} \right..$$

Finally, the STD-pooling matrix $M$ is formed by combining the two layers as shown below.

$$M=STD\left( n=48;q=7;k=2 \right)=\left[ \begin{matrix} \begin{matrix} L_{0} \\ L_{1} \end{matrix} \end{matrix} \right]=\left[ \begin{matrix} C_{0,0} & C_{1,0} & \ldots& C_{47,0} \\ C_{0,1} & C_{1,1} & \ldots& C_{47,1} \end{matrix} \right]$$

The matrix $M$ is transformed into S1A Table where all 48 kernel extracts are identified. The visualized matrix $M$ (S1 Fig) clearly shows the shifting pattern. The STD pooling scheme for 96 samples (n = 96; q = 5; k = 3) can be generated in the web-based app through this link: <https://echocharlie.shinyapps.io/STD_combined/>.

# References

Kainkaryam, R. M., & Woolf, P. J. (2008). poolHiTS: A Shifted Transversal Design based pooling strategy for high-throughput drug screening. *BMC Bioinformatics*, *9*, 256. https://doi.org/10.1186/1471-2105-9-256

Thierry-Mieg, N. (2006). A new pooling strategy for high-throughput screening: The Shifted Transversal Design. *BMC Bioinformatics*, *7*, 28. https://doi.org/10.1186/1471-2105-7-28
