# Supplementary material for: When to use one-dimensional, two-dimensional, and Shifted Transversal Design pooling in mycotoxin screening
Source: PLoS One. 2020 Aug 5;15(8):e0236668. doi: 10.1371/journal.pone.0236668 (PMC7406063; doi:10.1371/journal.pone.0236668)
Supplement: Attachment — Submitted filename: Review Response.docx [file pone.0236668.s010.docx]

**Reviewers' comments**

Reviewer's Responses to Questions

**Comments to the Author**

1. Is the manuscript technically sound, and do the data support the conclusions?

The manuscript must describe a technically sound piece of scientific research with data that supports the conclusions. Experiments must have been conducted rigorously, with appropriate controls, replication, and sample sizes. The conclusions must be drawn appropriately based on the data presented.

Reviewer #1: No

Reviewer #2: Partly

2. Has the statistical analysis been performed appropriately and rigorously?

Reviewer #1: I Don't Know

Reviewer #2: I Don't Know

3. Have the authors made all data underlying the findings in their manuscript fully available?

The [PLOS Data policy](http://www.plosone.org/static/policies.action#sharing) requires authors to make all data underlying the findings described in their manuscript fully available without restriction, with rare exception (please refer to the Data Availability Statement in the manuscript PDF file). The data should be provided as part of the manuscript or its supporting information, or deposited to a public repository. For example, in addition to summary statistics, the data points behind means, medians and variance measures should be available. If there are restrictions on publicly sharing data—e.g. participant privacy or use of data from a third party—those must be specified.

Reviewer #1: Yes

Reviewer #2: Yes

4. Is the manuscript presented in an intelligible fashion and written in standard English?

PLOS ONE does not copyedit accepted manuscripts, so the language in submitted articles must be clear, correct, and unambiguous. Any typographical or grammatical errors should be corrected at revision, so please note any specific errors here.

Reviewer #1: Yes

Reviewer #2: Yes

5. Review Comments to the Author

Review Comments to the Author

Please use the space provided to explain your answers to the questions above. You may also include additional comments for the author, including concerns about dual publication, research ethics, or publication ethics. (Please upload your review as an attachment if it exceeds 20,000 characters)

**Reviewer #1:** This manuscript attempts to address the question of whether and in what conditions pooling strategies are beneficial in the context of medium-scale experiments, taking into account the costs of pipetting from building the pools in addition to the costs of the assays themselves. This is a very interesting practical question. Unfortunately the manuscript fails to provide useful answers due to methodological flaws.

The main issue is that the pools used are inappropriate for the conditions considered in the manuscript. Indeed, when performing experiments on pools, the pool size should obviously depend on the expected number of positive samples, ie on the prevalence. For example with a prevalence of 35% as in Table 4 for fumonisin, any pool of 8 samples has 1 - 0.65^8 = 97% chance to contain a positive sample. Therefore building and testing such pools (eg 1D Row in Table 4) hardly provide any information at all: virtually every pool will be positive, hence each sample will need to be individually re-tested. There is no need for any kind of simulation or experiment to establish this.
Similarly, in this study the pool size of STD designs is set once and for all assuming a single contaminated sample (as explained P6), corresponding to a prevalence of 2% in 48-well format and 1% in 96-well format. The authors then use these pools in high prevalence situations, which obviously cannot work for the same reasons outlined above: every pool will be positive and nothing is learned from the pooling results.

Response: We made major changes to the “simulation of pooling” section (line 154-164) to clarify the methodology and we added a sentence at the end to respond to the concern of including high prevalence in the simulation. As for Table 4, it is intended to demonstrate the simulation can faithfully describe the pooling process and yield results comparable to empirical ones, including scenarios of high prevalence. From this perspective, we would argue that Table 4 serves the purpose at least partially. We admit that it would be better to validate the simulation with low-prevalence experimental samples, but we were unable to do it due to limited samples and reagent.

Although the exact number of positives is not known, one can usually have a rough estimate, and this is sufficient to design informative pools. For 1D and 2D designs the experimenter has limited control and can only choose the assay format (eg 48 or 96). However with STD designs the pool size can be set as desired by choosing the value of q. Given the expected prevalence, the standard procedure is to choose q such that approximately 50% of pools will be positive, while respecting the maximum pool size due to the assay LOD. For example, when testing aflatoxin contamination (expecting ~13% prevalence) one should aim for pools of size 5, therefore in 96 format a reasonable design could be STD(96;19;2). Testing such a design could provide interesting results, contrary to STD(96;5;3).

Response: We added a definition for q in line 203 – 205, clarifying it is the number of pools per layer instead of the pool size; the pool size is not directly controlled by the user but by the STD formation algorithm. We modified table 1 to present the STD pool sizes and added a sentence in line 204-208 to explain our STD choices.

Assuming 13% prevalence, neither STD(96; 19; 2) nor STD(96; 5; 3) is qualified according to the algorithm. Under this prevalence, it is STD(96; 17; 14) that requires the least number of tests (238 tests). Assuming 1% prevalence, STD(96; 5; 3) and STD(96; 19; 2) are both valid pooling schemes. However, STD(96; 5; 3) would require only 15 tests while STD(96; 19; 2) would require 38 tests. From an experimentalist perspective, one of the major goals of pooling is to save on reagent and reduce the amount of human labor. Therefore, while STD(96; 19; 2) may produce more interesting results, it may contradict our motivation to conducting pooling.

Other less critical issues follow.
- The manuscript is somewhat confusing between simulations and experiments. Could the authors please clarify what was studied through simulation and what exactly was tested experimentally. A figure representing the study design would be helpful for this.

Response: We created a flow chart (Fig.1) and added a summary paragraph in line 88-99 to clarify the study design.

- Concerning STD pools with n=48, the manuscript mostly uses STD(48;7;2) (eg Table 3) but in some places (eg Table 4) it uses STD(48;7;4) instead. Please clarify.

Response: We modified the paragraph at line 297-301 to clarify the rationale. We used STD(48; 7; 2) in all the simulations because it represents the lowest-prevalence case for 48-well plates. However, we used STD(48; 7; 4) in the validation experiment (Table 4) because the corn sample we used had a prior prevalence of 6%. At this prevalence, STD(48;7;4) requires the least number of tests and was thus selected. To validate STD(48; 7;4), we updated our simulation inputs and iterated 10000 times.

- Table 3 presents formulae for the number of pipetting, followed by Min and Max columns which should match, but they do not. For example the 1D Column and 1D Row formulas are identical, this must be wrong. Also the formulae for STD do not match the Min/Max values at all.

Response: We fixed the formulae in table 3 to correctly reflect what was implemented in the simulation program. Also, we added a sentence in line 247-248 and a footnote to the table to clarify that the min and max columns in table 3 contain observations in the simulation, not theoretical ones. While it does present a few results in the methods section, we feel this is the best way to allow the reader to both understand the methods of the paper and directly compare the theoretical and empirical values.

- The manuscript describes a method for interpreting the STD pooling results based on 2 criteria (P7). This method marks samples as positive or negative, and also leaves some samples unmarked. When every pool is positive this will result in every sample remaining unmarked, therefore there should be zero false positives when prevalence is high (contrary to the presented results). I suspect the authors apply a third criteria, where unmarked samples are considered positive? If so, it needs to be explained and justified.

Response: We revised line 216-221 to clarify the logical elimination rules and we added an example. In response to your question, we followed the two criteria stringently and did not apply a third criteria. Following the criteria, there should be 0 false negative but $\geq0$ false positives, assuming that we respect the maximum pool size and that the assay is reliable.

**Reviewer #2:** The topic of the paper is original and gives a theoretical and experimental study on different sample pooling techniques for mycotoxin screening through ELISA. It is fluently written. Nevertheless I have major questions and comments which I would like to be clarified.

First of all, a change in the title is needed: instead of detection, ‘screening’ is preferred, as this type of pooling only works for a screening technique with a dedicated cut-off level.

Response: We changed the title as advised.

The abstract should make clear from the beginning that the focus of the work is on ELISA (this is only mentioned in the middle of the abstract). It is also better to write one-dimensional (1D) and two-dimensional (2D) in line 18.

Response: We modified the abstract as advised.

GENERAL MAJOR COMMENTS

A major comment that I have on the paper is that a generalization is made that positive samples in mycotoxin analysis are rare (eg in the Introduction line 44). This however depends on the legislative maximum limits used as well on the region. Indeed, in line 67 you write that aflatoxin contamination of corn kernels is a low-prevalence event. I cannot agree that this generalization is made; eg in some regions in Africa this is a very high-prevalence event. This should be more diversified in the paper.

Response: We modified the sentence in line 68-71 to limit it to the U.S. and added a sentence (line 71-74) to mention high prevalence in other regions.

Another major comment is on the 20 ppb standard which is set by the FDA. As you know in other parts of the world, such as the European Union, maximum limits for aflatoxins are much lower; in that event, the pooling concept will probably not work, as the detection limit of ELISA might not be sensitive enough. It will be needed to discuss this in the paper, so that it can be interesting for broader set of readers.

Response: We modified the sentence in line 181-184 to explain how to set up pooling for a different regulatory level. We also included sentences in line 419-426 to explain how pooling may still work for different regulatory limits and different LODs.

Another major comment concerns that the study is based on the analysis of single kernels. This is not how routine control for mycotoxins in corn is generally performed. In order to have representative samples, corn lots are ground and different subsamples are analysed. So, I am really not convinced that the presented pooling can work in that case? As you work with representative samples, you won’t have those extremely high mycotoxin concentrations as you had in the study in one single kernel (as mentioned on p17, line 337: 4.0 x 104 ppb for aflatoxin B1 and 36 ppm for fumonisins). Please also have this in the discussion section.

Response: We added a paragraph (line 436-447) to address this.

Another major comment is on the contamination of the single corn kernels; on line 439 you write “we contaminated single corn kernels with different level of aflatoxin”. So, these were not naturally contaminated? And what about the fumonisins? Of course, if you work with spiked samples, this will also have an influence on the extraction, and will perhaps lead to much better reproducible results than with naturally contaminated ones. If you spiked yourself, you need to mention the method how this was done. On lines 139-141 you mention low-aflatoxin class, medium and high; where I had the impression that these were naturally contaminated; so a lot of confusion which needs to be cleared.

Response: In line 497-499, we modified the sentence to clarify that numbers were drawn from the fitted distributions to simulate contaminated kernels.

SPECIFIC COMMENTS

In the Materials and Methods section, it is needed to mention the Helica ELISA test which is used, more in the beginning of the description, so that it is clear that an ELISA with 1 ppb LOD has been used for the whole study.

Response: We added the LOD explanation in line 114-116 and 127-128.

I am also wondering that on p7, line 125, for the 96-well the positive-pool threshold is set at the LOD. So, what about false results when working at the LOD?

Response: We revised line 431-434 to warn about the possibility of having unreliable assay results when working at the LOD in reality.

In Line 135, suddenly also fumonisins are mentioned, while before only aflatoxins were mentioned; better to make clear from the start that both mycotoxin groups are part of the study.

Response: We brought the “experimental mycotoxin data” sub-section to the beginning of the method section. We also added a sentence in line 281-283 to explain that both aflatoxin and fumonisin were used to validate the pooling simulation because of limited reagent and samples.

Line 206: what is this ‘maximum limit of ELISA’?

Response: We deleted this phrase and modified the sentence (line 243 -245) to avoid confusion. In real experiments, if a sample exceeds the maximum limit in the standard curve, we will dilute the sample, re-test it, and repeat these two steps until it falls within the allowable range. Given the arbitrariness in sample dilution, we decided not to include these further re-tests in the simulation.

I was wondering why the 1D and 2D pooling strategies were only validated with fumonisins and the STD only with aflatoxin?

Response: We added a sentence in line 281-283 to explain that we only had limited sample extracts and ELISA kits. After we validated the STD pooling with aflatoxin, we ran out of both the samples and the aflatoxin ELISA reagent. Thus, we had to validate the other pooling strategies with new samples and fumonisin ELISA kit.

Line 261: why only >20 ppb aflatoxin is mentioned, and nothing about fumonisin?

Response: We put the fumonisin limit in line 125-127.
